# Measurement of Thromboxane Biosynthesis in Health and Disease

**DOI:** 10.3389/fphar.2019.01244

**Published:** 2019-10-30

**Authors:** Carlo Patrono, Bianca Rocca

**Affiliations:** Department of Pharmacology, Catholic University School of Medicine, Rome, Italy

**Keywords:** thromboxane, prostanoids biosynthesis, aspirin, cardiovasular disease, platelet activation

## Abstract

Thromboxane (TX) A_2_ is a chemically unstable lipid mediator involved in several pathophysiologic processes, including primary hemostasis, atherothrombosis, inflammation, and cancer. In human platelets, TXA_2_ is the major arachidonic acid derivative *via* the cyclooxygenase (COX)-1 pathway. Assessment of platelet TXA_2_ biosynthesis can be performed *ex vivo* through measurement of serum TXB_2_, an index of platelet COX-1 activity, as well as *in vivo* through measurement of urinary enzymatic metabolites, a non-invasive index of platelet activation. This article reviews the main findings of four decades of clinical investigation based on these analytical approaches, focusing on the measurement of TXA_2_ metabolites to characterize the pathophysiologic role of transiently or persistently enhanced platelet activation and to describe the clinical pharmacology of COX-1 inhibition in health and disease.

## Introduction

**Figure 1:**
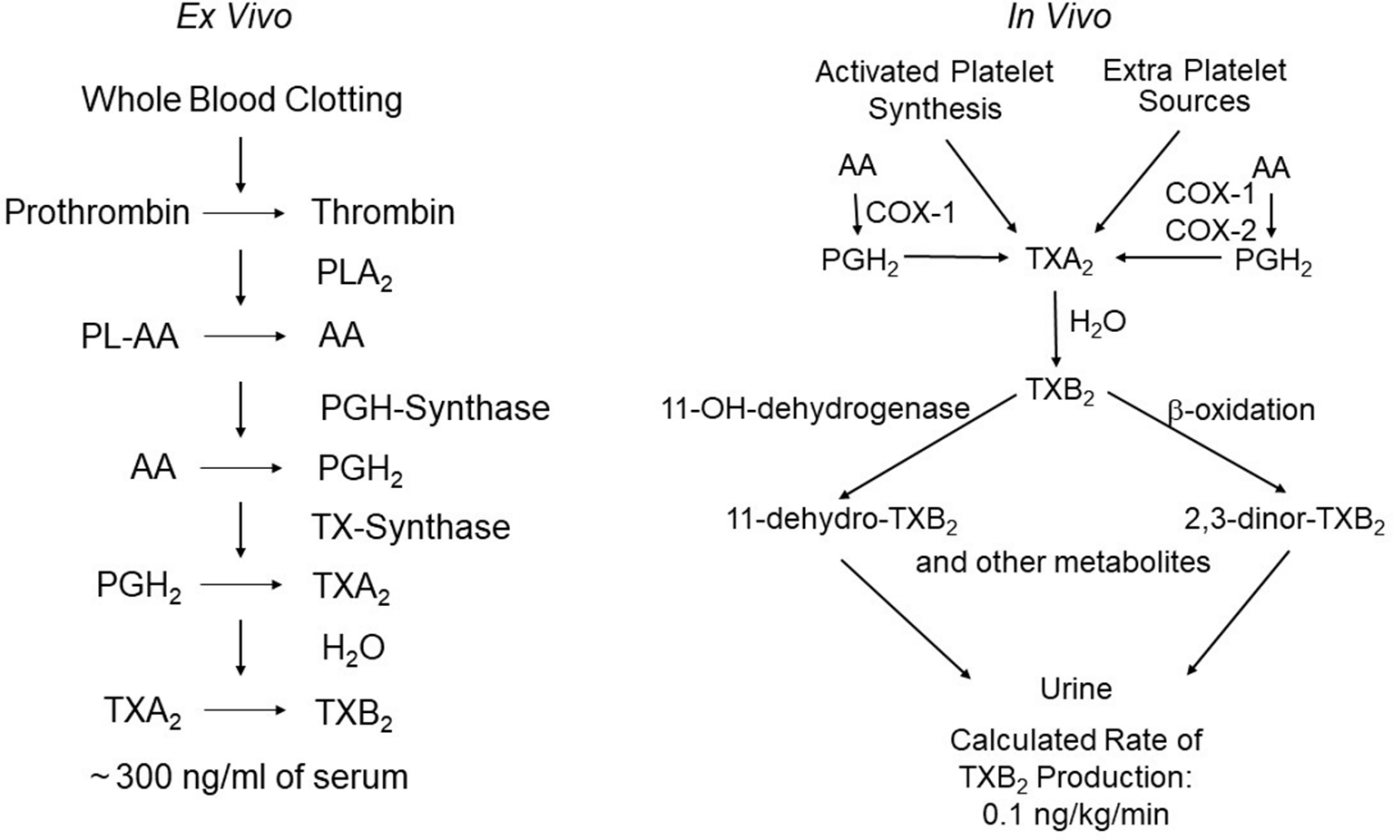
Assessment of platelet thromboxane (TX)A_2_ biosynthesis *ex vivo* and *in vivo*. The left panel depicts the chain of enzymatic reactions triggered by thrombin generation during whole-blood clotting, and the resulting formation of very large amounts of TXB_2_ (the stable hydration product of TXA_2_) that can be easily measured in serum as a sensitive and specific index of platelet COX-1 activity. The right panel depicts the metabolic fate of TXA_2_*in vivo* and the calculated rate of its production in healthy subjects on the basis of TXB_2_ infusions and measurement of its major urinary metabolites, 11-dehydro-TXB_2_ and 2,3-dinor-TXB_2_. The latter represent a non-invasive index of platelet activation *in vivo*. Modified and redrawn from Patrono et al. (2013).

The pictorial description in 1962 of the aggregation of blood platelets by ADP, using a novel device called the Born aggregometer (Born, 1962), paved the way to quantitative assessment of platelet inhibition *in vitro* and *ex vivo* (reviewed by Born and Patrono, 2006). However, the possibility of establishing a mechanistic link between the inhibition of platelet prostanoid formation by aspirin (Smith and Willis, 1971) and inhibition of platelet aggregation had to wait the discovery of a novel pro-aggregating and vasoconstrictor prostanoid, thromboxane (TX)A_2_, as the major arachidonic acid derivative in human platelets (Hamberg et al., 1975). This discovery allowed the development of appropriate analytical tools to investigate platelet TXA_2_ biosynthesis and its inhibition by aspirin in human health and disease (reviewed by Born and Patrono, 2006). TXA_2_ is a pro-thrombotic, chemically unstable prostanoid, mostly synthesized *via* cyclooxygenase (COX)-1 and released by activated platelets (reviewed by Davì and Patrono, 2007). Two different biomarkers were characterized independently to assess TXA_2_ biosynthesis *ex vivo*, i.e., whole-blood TXB_2_ production (Patrono et al., 1980), and *in vivo*, i.e., urinary TXB_2_ metabolite excretion (Roberts et al., 1981) (**Figure 1**). By raising a specific antibody against TXB_2_, the chemically stable and biologically inactive hydrolysis product of TXA_2_, we were able to measure the time-dependent synthesis and release of platelet TXA_2_ induced by endogenous thrombin generated during whole-blood clotting and to demonstrate its suppression by low doses of aspirin (Patrono et al., 1980). The pioneering work of John Oates and his associates at Vanderbilt University was responsible for the development of a non-invasive approach to investigating prostanoid biosynthesis in man, based on the measurements of urinary prostanoid metabolites. In 1981, they reported the conversion of systemically infused TXB_2_ into 20 enzymatic derivatives, which were identified in the urine of a single healthy volunteer by gas chromatography/mass spectrometry (GC/MS) (Roberts et al., 1981). The availability of these analytical tools paved the way for investigating TXA_2_ biosynthesis in health and disease and its selective, cumulative inhibition by low-dose aspirin, that eventually led to its development as an antiplatelet drug for the treatment and prevention of atherothrombosis (reviewed by Patrono, 1994).

This article reviews the main findings of four decades of clinical investigation based on these analytical approaches, focusing on the measurement of TXA_2_ metabolites *in vivo* and *ex vivo* as indexes of platelet activation and COX-1 activity, respectively, with emphasis on the authors’ contribution to the resulting pathophysiological and pharmacological developments.

## Urinary Thromboxane Metabolite Excretion as a Non-Invasive Biomarker of Platelet Activation *In Vivo*

In 1981, Roberts et al. reported the GC/MS characterization of 20 enzymatic metabolites of systemically infused [^3^H_8_]TXB_2_. Two major series of metabolites were identified based on a ring structure. One series retained the original TXB_2_ hemiacetal ring and included two metabolites, 2,3-dinor-TXB_2_ (the most abundant urinary metabolite) and 2,3,4,5-tetranor-TXB_2_, which were products of beta-oxidation (Roberts et al., 1981). The second group of derivatives was formed as a result of dehydrogenation of the hemiacetal alcohol group at C-11, and included 16 metabolites (Roberts et al., 1981). Among the compounds resulting from this single transformation, 11-dehydro-TXB_2_ was the most abundant urinary metabolite (Roberts et al., 1981). In their seminal paper in the *Journal of Biological Chemistry*, Roberts et al. accurately predicted the potential value of this analytical approach: “Since thromboxanes are released in substantial quantities from aggregating platelets, quantification of *in vivo* thromboxane production may provide a means to assess *in vivo* platelet aggregation and lead to a better understanding of the role of platelets in the pathophysiology of many cardiovascular diseases. It may also provide a means to assess the *in vivo* efficacy of anti-platelet drug therapy” (Roberts et al., 1981). Important limitations of this study were represented by a single high rate of TXB_2_ infusion and a single healthy subject being infused, precluding assessment of the linearity of conversion of TXB_2_ into its major enzymatic derivatives, as well as of the interindividual variability in the prevalence of the two main pathways of its metabolic transformation.

Together with Garret FitzGerald and Ian Blair, we reexamined the metabolic fate of TXB_2_ entering the systemic circulation, by measuring the urinary excretion of 2,3-dinor-TXB_2_ during the infusion of exogenous TXB_2_, in four aspirin-pretreated healthy volunteers randomized to receive 6-h i.v. infusions of vehicle alone and TXB_2_ at 0.1, 1.0, and 5.0 ng·kg^−1^·min^−1^ (Patrono et al., 1986). Plasma TXB_2_ and urinary 2,3-dinor-TXB_2_ were measured before, during, and up to 24 h after the infusions and in aspirin-free periods. Aspirin treatment suppressed baseline urinary 2,3-dinor-TXB_2_ excretion by 80%, consistent with a predominant platelet source of the parent compound. The fractional excretion of 2,3-dinor-TXB_2_ was independent of the rate of TXB_2_ infusion, over a 50-fold dose range, and averaged 5.3% ± 0.8% (Patrono et al., 1986). Insertion of 2,3-dinor-TXB_2_ excretion rates measured in aspirin-free periods into the linear relationship between the doses of infused TXB_2_ and the amounts of metabolite excreted in excess of control values permitted estimation of the rate of entry of endogenous TXB_2_ into the circulation as 0.11 ng·kg^−1^·min^−1^ (Patrono et al., 1986). Upon discontinuing TXB_2_ infusion, its rate of disappearance from the systemic circulation was linear over the first 10 min with an apparent half-life of 7 min. This resulted in a maximal estimate of the plasma concentration of endogenous TXB_2_ of 2.0 pg/ml, i.e., much lower than had been previously reported (Patrono et al., 1986). This finding argued for a local nature of TXA_2_ synthesis and action, as previously suggested for prostacyclin (PGI_2_) (FitzGerald et al., 1981). Similar to the endothelial synthesis of PGI_2_, the maximal TXA_2_ biosynthetic capacity of human platelets greatly exceeds its actual production *in vivo*. Thus, the platelets of 1 ml of whole blood clotted for 1 h *in vitro* can synthesize and release a similar amount of TXB_2_ as that secreted into the systemic circulation *in vivo* during the same time (Patrono et al., 1980; Patrono et al., 1986) (**Figure 1**), a finding that may help explain the unusual requirement for greater than 97% inhibition of TXA_2_ biosynthetic capacity to maximally inhibit TXA_2_-dependent platelet function (Reilly and FitzGerald, 1987; Santilli et al., 2009) (see below).

However, because of obvious safety concerns, it had not been possible to investigate the metabolic fate of TXA_2_ in humans, and it remained to be determined whether the enzymatic transformation of TXB_2_ to its major urinary metabolites accurately reflected TXA_2_ metabolism *in vivo*. Thus, together with Joe Rokach and his colleagues at Merck Frosst Research Laboratories, Patrignani et al. (1989) compared the metabolic handling of exogenously infused TXA_2_ and TXB_2_ in the cynomolgus monkey. The main finding of this study was that TXA_2_ and TXB_2_ are metabolized to 2,3-dinor-TXB_2_ and 11-dehydro-TXB_2_ with similar fractional conversion rates, thereby suggesting that TXA_2_ is hydrolyzed non-enzymatically to TXB_2_ prior to enzymatic degradation *via* the beta-oxidation and 11-OH-dehydrogenase pathways, and that the resulting urinary metabolites provide a quantitative index of TXA_2_ biosynthesis *in vivo* (Patrignani et al., 1989).

Because previous estimates of the rate of entry of TXB_2_ into the human systemic circulation had been based on monitoring the beta-oxidation pathway of TXB_2_ metabolism (Patrono et al., 1986), Ciabattoni et al. (1989) went on to measure the urinary excretion of immunoreactive 11-dehydro-TXB_2_ and 2,3-dinor-TXB_2_ (Ciabattoni et al., 1987) during the infusion of exogenous TXB_2_ over a 50-fold dose range in healthy volunteers, with the same protocol of the previous study (Patrono et al., 1986). The fractional elimination of both metabolites was independent of the rate of TXB_2_ infusion and averaged 6.0% to 7.0%, demonstrating that urinary 11-dehydro-TXB_2_ is at least as abundant a conversion product of exogenously infused TXB_2_ as 2,3-dinor-TXB_2_ (Ciabattoni et al., 1989) Furthermore, the study of Ciabattoni et al. (1987) showed that this analytical approach could detect changes in the urinary excretion of immunoreactive 11-dehydro-TXB_2_ associated with simulated short-term increases of TXB_2_ release into the human circulation (Ciabattoni et al., 1987).

Transient increases in the excretion of 2,3-dinor-TXB_2_ and 11-dehydro-TXB_2_ were described in patients with acute coronary syndromes and interpreted as reflecting repeated episodes of platelet activation (Fitzgerald et al., 1986; Vejar et al., 1990). Transient changes in TXA_2_ biosynthesis detected in patients with unstable angina were accompanied by concomitant increases in PGI_2_ biosynthesis, as reflected by urinary 2,3-dinor-6-keto-PGF_1α_ excretion, suggesting a counter-regulatory endothelial activation in this setting (Fitzgerald et al., 1986). In contrast, patients with chronic stable angina did not display increased TXA_2_ biosynthesis, both under resting conditions and following exercise-induced myocardial ischemia (Fitzgerald et al., 1986). Biochemical evidence of episodic platelet activation in the setting of acute coronary syndromes was consistent with the post-mortem findings of Michael Davies and Erling Falk that suggested dynamic thrombotic events occurring over a disrupted plaque in the coronary vessels of patients dying after a diagnosis of unstable angina (reviewed by Falk et al., 1995; Davì and Patrono, 2007). These results provided a rationale for testing the efficacy and safety of low-dose aspirin in acute coronary syndromes, a clinical setting in which antiplatelet therapy reduced the risk of major atherothrombotic complications by approximately 50% (reviewed by Patrono et al., 2005). Episodic increases in TXB_2_ metabolite excretion were also characterized in patients with acute ischemic stroke (Koudstaal et al., 1993; van Kooten et al., 1997), though with a lower frequency and shorter duration than in acute coronary syndromes (Fitzgerald et al., 1986; Vejar et al., 1990), perhaps reflecting the heterogeneity of mechanisms responsible for ischemic stroke (Albers et al., 2004). Low-dose aspirin (50 mg daily) largely suppressed 11-dehydro-TXB_2_ excretion in this setting, reflecting the predominant platelet origin of TXA_2_ biosynthesis (Koudstaal et al., 1993). Enhanced platelet activation was independently associated with stroke severity on admission (van Kooten et al., 1997). Patients with a transient ischemic attack were characterized by infrequent episodes of platelet activation, suggesting that enhanced TXA_2_ biosynthesis was not secondary to cerebral ischemia (Koudstaal et al., 1993).

Persistent platelet activation, as reflected by persistently enhanced urinary excretion of TXB_2_ metabolites, was reported by different Groups in patients with a variety of cardiovascular risk factors that accelerate atherogenesis, including cigarette smoking (Nowak et al., 1987), type-2 diabetes mellitus (Davì et al., 1990), hypercholesterolemia (Davì et al., 1992), homozygous homocystinuria (Di Minno et al., 1993), and hypertension (Minuz et al., 2002). Because enhanced urinary excretion of 11-dehydro-TXB_2_ in diabetes mellitus might reflect either an abnormality in the biosynthesis of TXA_2_ or a shift in its metabolic fate through the two main enzymatic pathways of degradation, Davì et al. (1990) investigated the fractional conversion of infused TXB_2_ to urinary 11-dehydro-TXB_2_ in subjects with type-2 diabetes mellitus. Their finding of a linear conversion of infused TXB_2_ to urinary 11-dehydro-TXB_2_ over a 50-fold range of infusion rates, with a fractional elimination similar to that previously described in healthy subjects (Ciabattoni et al., 1989), was consistent with enhanced excretion of 11-dehydro-TXB_2_ reflecting a change in the biosynthesis of TXA_2_ rather than an alteration in its enzymatic transformations (Davì et al., 1990).

Urinary prostanoid metabolites, such as 11-dehydro-TXB_2_, do not reflect a specific site of prostanoid biosynthesis. To characterize the potential platelet versus non-platelet sources of TXA_2_ biosynthesis, Davì et al. (1990) used the unique property of aspirin to produce selective, cumulative acetylation of platelet cyclooxygenase (COX)-1 when it is given in low doses once daily (Patrignani et al., 1982). Renal COX-isozymes, which have been involved in enhanced TXA_2_ production in patients with systemic lupus erythematosus (Patrono et al., 1985), are not inhibited by low-dose aspirin to any detectable extent (Patrignani et al., 1982; Pierucci et al., 1989). The profound reduction in urinary 11-dehydro-TXB_2_ excretion that was found in subjects with type-2 diabetes mellitus after they received 50 mg of aspirin daily for 1 week and the platelet turnover-dependent return to a pre-treatment excretion rate over the next 10 days following aspirin discontinuation were consistent with a prevailing role for platelets as the source of TXA_2_ biosynthesis in this setting (Davì et al., 1990).

**Figure 2:**
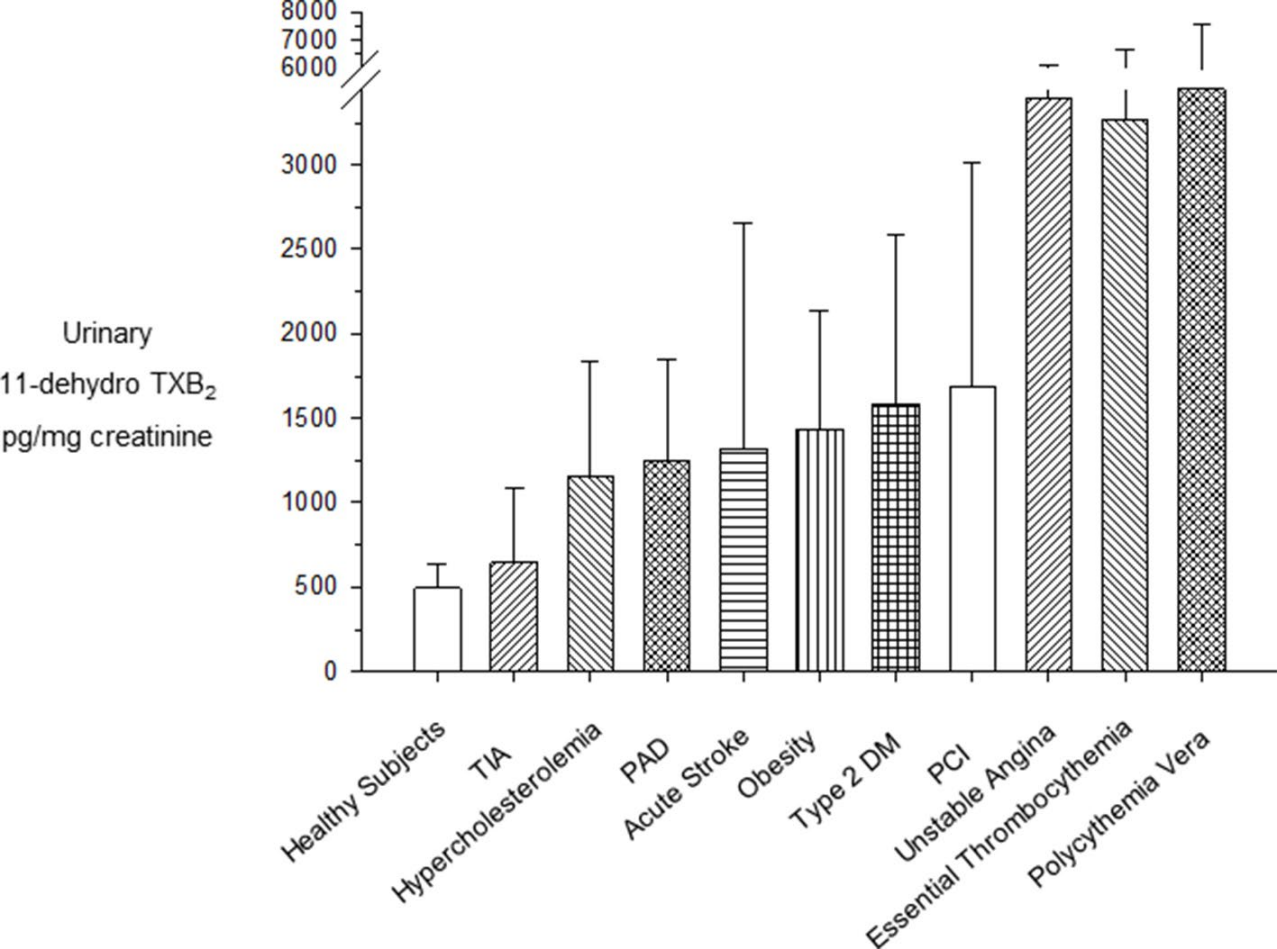
Urinary excretion rates of 11-dehydro-TXB_2_ in clinical settings at high cardiovascular risk. Mean (± standard deviation) or median (interquartile range) urinary excretion rates of 11-dehydro-TXB_2_ in clinical settings characterized by high cardiovascular risk. PAD, peripheral arterial disease; PCI, percutaneous coronary intervention; T2DM, type 2 diabetes mellitus; TIA, transient ischemic attack. Modified and redrawn from Patrono et al. (2013).

Persistently enhanced urinary excretion of TXB_2_ metabolites has been reported in the vast majority of patients with myeloproliferative neoplasms, such as essential thrombocythemia (ET) and polycythemia vera (PV) (reviewed by Patrono et al., 2013). As shown in **Figure 2**, the urinary excretion rates of 11-dehydro-TXB_2_ measured in untreated ET (Rocca et al., 1995) and PV patients (Landolfi et al., 1992) are comparable to the rate measured in patients with unstable angina (Fitzgerald et al., 1986; Vejar et al., 1990) and higher than values of metabolite excretion associated with a variety cardiovascular risk factors (Nowak et al., 1987; Davì et al., 1990; Davì et al., 1992; Di Minno et al., 1993; Minuz et al., 2002). The finding of persistently enhanced TXA_2_ biosynthesis in PV patients and its suppression by low-dose aspirin (Landolfi et al., 1992) provided a rationale for testing the efficacy and safety of this antiplatelet strategy in PV (Landolfi et al., 2004). Based on the positive results of the ECLAP (European Collaboration on Low-dose Aspirin in Polycythemia vera) trial (Landolfi et al., 2004), primary prophylaxis with low-dose aspirin (81–100 mg daily) is currently recommended for PV patients (Tefferi et al., 2018).

Urinary 11-dehydro-TXB_2_ excretion has been investigated as a potential biomarker of the future risk of major vascular events in aspirin-treated, high-risk patients enrolled in the Heart Outcomes Prevention Evaluation (HOPE) trial, by using a nested case-control design (Eikelboom et al., 2002). After adjustment for baseline differences, the odds for the composite end-point of myocardial infarction, stroke, or cardiovascular death increased with each increasing quartile of baseline urinary 11-dehydro-TXB_2_, with patients in the upper quartile having approximately 2-fold higher risk than those in the lower quartile (Eikelboom et al., 2002). This finding was largely confirmed by a similar substudy of the Clopidogrel for High Atherothrombotic Risk and Ischemic Stabilization, Management and Avoidance (CHARISMA) trial (Eikelboom et al., 2008). However, because both HOPE and CHARISMA were mostly secondary prevention trials, an important limitation of these analyses is represented by the lack of a control group of subjects not treated with aspirin. Furthermore, the extent of biological variation in urinary 11-dehydro-TXB_2_ excretion rate, as well as the intrasubject variability in its reduction by low-dose aspirin are currently unknown, but could potentially limit the value of this biomarker to predict the risk of future cardiovascular events in an individual aspirin-treated patient. We are currently investigating the potential predictive value of urinary 11-dehydro-TXB_2_ excretion in a large sample of A Study of Cardiovascular Events in Diabetes (ASCEND) that randomized 15,480 adults with diabetes mellitus to long-term treatment with low-dose aspirin or placebo (ASCEND Study Collaborative Group, 2018).

Recently, the platelet origin of urinary thromboxane metabolite (TXM) excretion was challenged by the case report of a single patient with end-stage renal failure requiring dialysis who carried a rare genetic mutation in cPLA_2α_ (cytosolic phospholipase A_2_), resulting in dramatically reduced urinary TXM and PGI_2_ metabolite (PGIM) excretion rates, with recovery of “normal” urinary levels of these prostanoid metabolites after kidney transplantation (Mitchell et al., 2018). The authors’ conclusion was that “urinary PGIM and TXM can be derived exclusively by the kidney without contribution from PGI_2_ made by endothelial cells or TXA_2_ by platelets in the systemic circulation” (Mitchell et al., 2018). However, the authors did not investigate the metabolic disposition of TXB_2_ and PGI_2_ in this patient, and no comparison was performed with renal failure and transplanted patients without the cPLA_2α_ genetic defect (Grosser et al., 2018). Moreover, it should be emphasized that in patients with systemic lupus erythematosus, who displayed enhanced renal synthesis of TXA_2_, higher urinary TXB_2_ excretion was associated with unchanged urinary excretion of 2,3-dinor-TXB_2_ (Patrono et al., 1985), suggesting that TXA_2_ produced by the kidney is mostly excreted unchanged into the urine and does not undergo systemic metabolism to TXM (Remuzzi et al., 1992).

## Serum TXB_2_ as a Validated Index of Platelet COX-1 Activity

**Figure 3:**
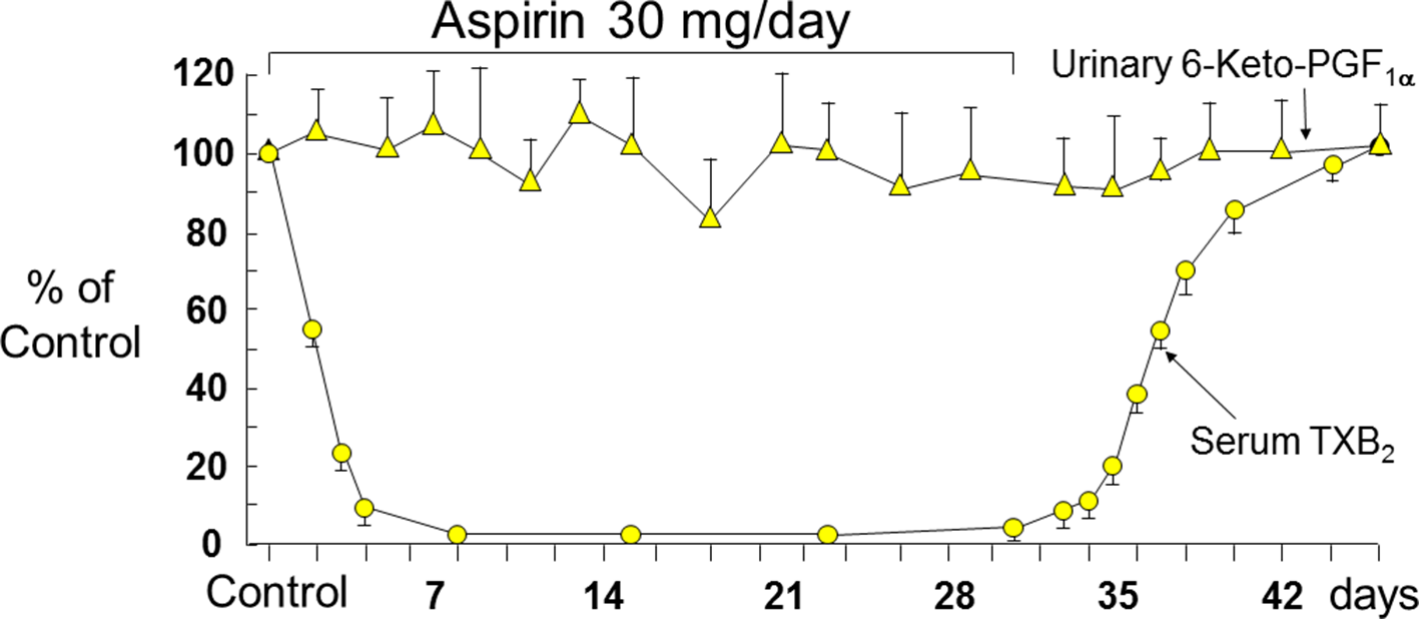
Long-term effects of low-dose (0.45 mg/kg per day) aspirin on platelet TXB_2_ and renal PGI_2_ synthesis. Serum TXB_2_ concentrations and urinary excretion of 6-keto-PGF_1α_ were measured in three healthy subjects before, during, and after aspirin therapy. Mean values ± SEM are plotted as percentage of control measurements performed prior to aspirin administration. The arrows indicate duration of daily aspirin therapy. Redrawn from Patrignani et al. (1982).

The effects of aspirin on the activity of platelet COX-1 have been investigated through measurements of serum TXB_2_ (Patrono et al., 1980; Patrignani et al., 1982) and urinary metabolites of TXB_2_ (FitzGerald et al., 1983). Serum TXB_2_ reflects the maximal biosynthetic capacity of blood platelets to generate TXA_2_ in response to endogenously formed thrombin during whole-blood clotting, and its measurement has been used extensively to assess the human pharmacology of platelet COX-1 inhibition in health and disease (reviewed by Patrono et al., 2008). Three important features of aspirin pharmacodynamics were characterized by measurements of serum TXB_2_ in healthy subjects (Patrignani et al., 1982): i) the cumulative nature of the inactivation of platelet COX-1 by repeated daily low doses (20–40 mg) of aspirin; ii) the saturability of this effect with single doses as low as 100 mg; and iii) the relative selectivity for platelet COX-1 inhibition at low doses. Our Research Group at the Catholic University School of Medicine in Rome described time-dependent, cumulative reduction in serum TXB_2_ (a product of platelet COX-1 activity), without statistically significant changes in urinary 6-keto-PGF_1α_ excretion (mostly a product of renal COX-2 activity), a reverse paradigm of selective COX-isozyme inhibition by low-dose aspirin (30 mg daily) in man (Patrignani et al., 1982) (**Figure 3**). Measurements of serum TXB_2_ were also instrumental in characterizing the presystemic nature of platelet COX-1 acetylation by aspirin, an important feature of aspirin pharmacodynamics contributing to its biochemical selectivity (Pedersen and FitzGerald, 1984). Thus, serum TXB_2_ was reduced by about 40% five min after the oral administration of a 20-mg dose, before aspirin could be detected in the systemic circulation (Pedersen and FitzGerald, 1984).

It is important to realize that the serum TXB_2_ assay requires almost immediate whole-blood incubation at 37°C as a condition for optimal thrombin generation, arachidonic acid release and its sequential conversion by platelet COX-1 and TX-synthase to form PGH_2_ and TXA_2_, respectively. As a consequence of thrombin-induced platelet activation, the blood concentration of TXB_2_ increases over 60 min from 1 to 2 pg/ml (true circulating plasma level *in vivo*) to 300 to 400 ng/ml (maximally stimulated, COX-1-dependent production *ex vivo*) (Patrono et al., 1980). A variably delayed access to 37°C incubation, as well as different analytical methods to quantitate serum TXB_2_, may contribute to variable results in a multicenter setting (Frelinger et al., 2009; Reny et al., 2012). Petrucci et al. (2016) investigated whether a variable delay in 37°C incubation and/or analytical discrepancies may affect the assessment of aspirin pharmacodynamics based on serum TXB_2_ determinations. They found that a longer than 5-min delay in the 37°C incubation of whole-blood samples may variably influence the assessment of platelet COX-1 inhibition by low-dose aspirin and confound the analysis of aspirin responsiveness in the clinical setting (Petrucci et al., 2016; De Stefano et al., 2018). In contrast, a GC/MS-validated immunoassay and liquid chromatography-tandem mass-spectrometry yielded quite comparable TXB_2_ concentrations in the same serum samples (Petrucci et al., 2016).

**Figure 4:**
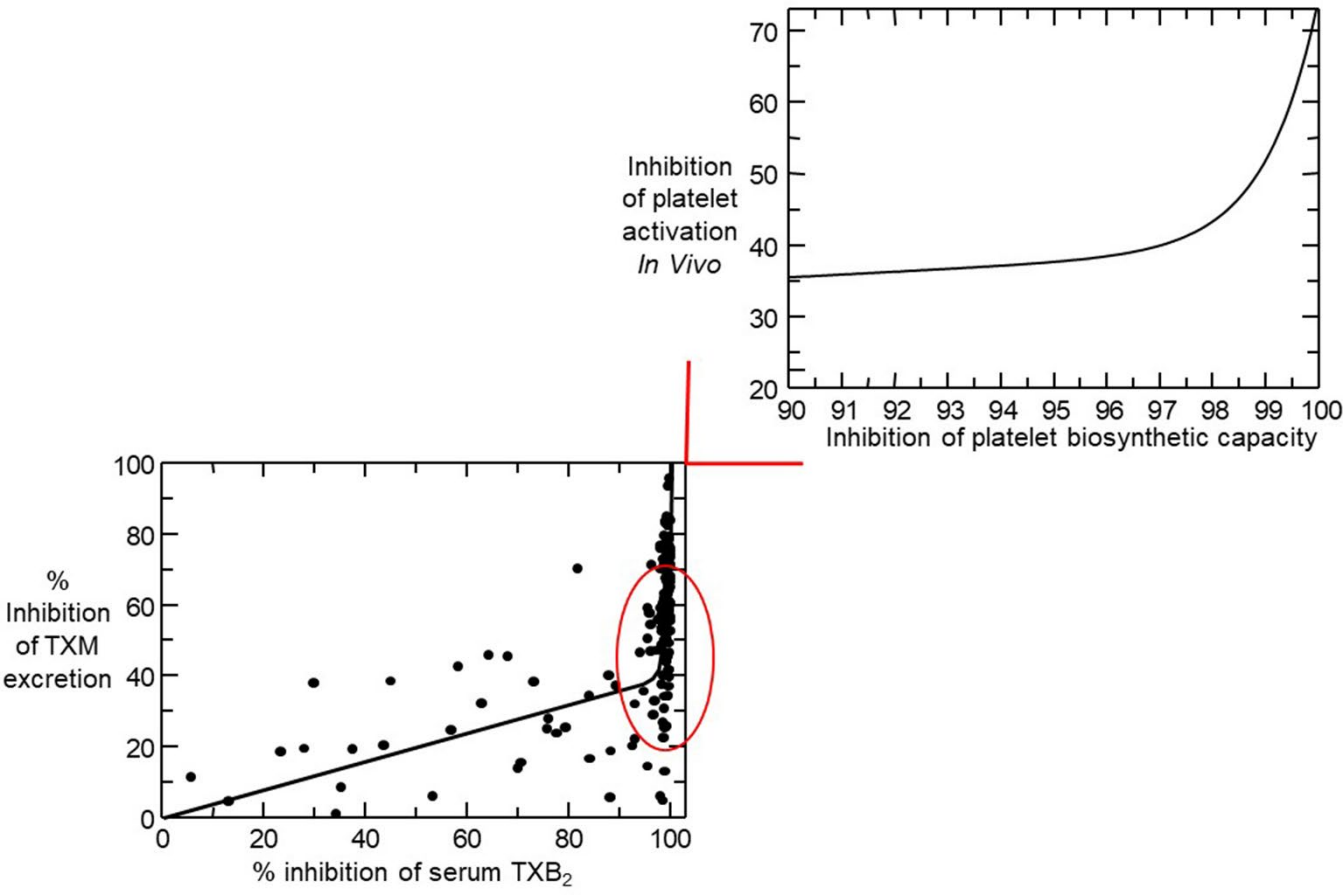
Nonlinear relationship between inhibition of serum thromboxane (TX) B_2_ and urinary 11-dehydro-TXB_2_ excretion. In the lower left panel, individual percentage inhibition values are depicted from all on-treatment and post-treatment measurements performed in 48 healthy subjects randomized to receive aspirin 100 mg daily for 1 to 8 weeks. The nonlinear relationship between percent inhibition of serum TXB_2_ and urinary 11-dehydro-TXB_2_ showed that for 0 to 97% of COX-1 inhibition, TXA_2_ biosynthesis *in vivo* was linearly inhibited by <40% and that >97% suppression of serum TXB_2_ was necessary to maximally reduce TX metabolite (TXM) excretion. The upper right panel represents a detail of the left panel and is based on mathematical modeling of the experimental data. Modified and redrawn from Santilli et al. (2009), with permission from the publisher.

The relationship between inhibition of platelet COX-1 activity, as reflected by serum TXB_2_, and arachidonic acid-dependent platelet function assays (i.e., arachidonate-induced optical aggregation), and urinary 11-dehydro-TXB_2_ excretion is strikingly non-linear (**Figure 4**) (Reilly and FitzGerald, 1987; Santilli et al., 2009). Thus, platelet COX-1 activity must be nearly completely (>97%) suppressed to fully inhibit *in vivo* platelet activation. Such stringent requirement may help explain the fact that the vast majority of traditional nonsteroidal antiinflammatory drugs (tNSAIDs), that are reversible inhibitors of platelet COX-1 with variable half-lives, are unable to achieve profound and persistent suppression of TXA_2_ biosynthesis, thereby unmasking their COX-2–dependent cardiovascular toxicity (reviewed by Patrono and Baigent, 2014). Although the variable COX-isozyme selectivity, half-life, daily dose, and duration of treatment of different COX-2 inhibitors could all influence the cardiovascular consequences of COX-2 inhibition, the dichotomous clinical read-outs of such inhibition are explained by the exponential relationship between inhibition of platelet COX-1 activity and suppression of TXA_2_-dependent platelet activation (**Figure 4**) (Patrono and Baigent, 2014). As depicted in **Figure 4**, NSAIDs inhibiting platelet COX-1 activity by 0% to 20% (e.g., highly selective COX-2 inhibitors, such as rofecoxib and etoricoxib), by 20% to 50% (e.g., COX-2 inhibitors with moderate COX-2 selectivity, such as diclofenac and celecoxib), or by 50% to 90% (most tNSAIDs, such as indomethacin and ibuprofen) will cause similarly modest suppression of TXA_2_-dependent platelet activation *in vivo* (Patrono and Baigent, 2014). Due to its longer half-life and modest COX-1 selectivity, naproxen 500 mg twice daily may suppress platelet COX-1 activity by >95% throughout the 12-h dosing interval and reduce TXA_2_-dependent platelet activation *in vivo* to a similar extent as aspirin 100 mg once daily (Capone et al., 2004; Capone et al., 2007).

Given the non-linear relationship depicted in **Figure 4**, platelet function assays, including urinary 11-dehydro-TXB_2_, do not accurately reflect the degree of platelet COX-1 inhibition (Pascale et al., 2012; Smith et al., 2012). Moreover, platelet function assays routinely used to measure aspirin response, are not necessarily related to its mechanism of action, display poor inter-assay agreement (Lordkipanidzé et al., 2007; Blais et al., 2009; Santilli et al., 2009; ), and give inconsistent results upon repeated measurements (Muir et al., 2009; Santilli et al., 2009). These methodological considerations may help explain the rise and fall of the aspirin “resistance” concept, typically defined as impaired platelet response to aspirin based on a single functional measurement performed at an often unspecified time point after dosing, usually without a reliable assessment of compliance (Michelson et al., 2005; Rocca and Patrono, 2005). In a study of 48 healthy volunteers, repeated measurements of platelet aggregation in response to different agonists demonstrated that occasionally “resistant” subjects could be classified as “responders” when examined previously or subsequently (Santilli et al., 2009). Not surprisingly, the incidence of “resistance” ranged from 1% up to 65% in different studies, was assay-dependent, fluctuated over time, and remains of unproven clinical significance (reviewed by Patrono and Rocca, 2008).

In contrast to the uniform effectiveness of low-dose aspirin in suppressing platelet COX-1 activity in healthy individuals, some clinical conditions are associated with transient or persistent suboptimal platelet inhibition by a conventional once daily regimen of low-dose aspirin (Rocca and Patrono, 2005). These include patients following on-pump coronary artery bypass surgery (CABG) (Cavalca et al., 2017), patients with ET (Dragani et al., 2010; Dillinger et al., 2012), patients with coronary artery disease and the metabolic syndrome (Smith et al., 2012), and some patients with type-2 diabetes mellitus (Spectre et al., 2011; Rocca et al., 2012; Bethel et al., 2016). Under these circumstances, most patients display biochemical evidence of TXA_2_-dependent platelet activation *in vivo* (Davì et al., 1990; Rocca et al., 1995), a finding that may help explain impaired aspirin pharmacodynamics. More specifically, less-than-optimal inactivation of platelet COX-1 could be a consequence of transiently (Cavalca et al., 2017) or persistently (Dragani et al., 2010) accelerated platelet renewal, or result from platelet activation-induced generation of hydroperoxides that may impair the acetylation of COX-1 by aspirin (Bala et al., 2008). The long-lasting duration of the antiplatelet effect of aspirin, despite its very short half-life, is explained by inactivation of COX-1 in bone-marrow platelet progenitors, as reflected by the 48-h delay between aspirin withdrawal and initial recovery of unacetylated COX-1 (Burch et al., 1978) and TXA_2_ biosynthetic capacity (Patrignani et al., 1982) in peripheral blood platelets. Thus, under conditions of normal thrombopoiesis, the efficacy of a short-lived drug given once daily reflects irreversible inactivation of a slowly renewable drug target (platelet COX-1) combined with an effect on bone-marrow platelet progenitors, leading to a new platelet progeny with largely non-functioning COX-1 for the vast majority of the 24-h dosing interval (Giaretta et al., 2017). However, reduced systemic bioavailability of aspirin, as may occur with some enteric-coated formulations (Maree et al., 2005) and in association with obesity (Petrucci et al., 2019), or faster renewal of platelet COX-1, as reported under conditions of altered megakaryopoiesis (Pascale et al., 2012), may shorten the duration of the antiplatelet effect of aspirin and dictate a more frequent dosing regimen (Patrono et al., 2013).

A standard once-daily regimen of low-dose aspirin administration cannot adequately suppress platelet TXA_2_ production, throughout the 24-h dosing interval, in the vast majority of ET patients (Dragani et al., 2010; Dillinger et al., 2012; Pascale et al., 2012). An accelerated turnover of platelet COX-1, reflecting abnormal megakaryopoiesis, has been hypothesized in ET (Pascale et al., 2012; Rocca and Patrono, 2015; Giaretta et al., 2017). A higher daily fraction of newly released platelets with unacetylated COX-isozymes would account for incomplete inhibition, as well as time-dependent recovery of TXA_2_-dependent platelet function during the standard 24-h dosing interval of low-dose aspirin administration (Dragani et al., 2010). Two relatively small studies in ET patients have shown that sub-optimal inhibition of platelet TXA_2_ production and TXA_2_-dependent platelet function can be largely rescued by a bid regimen of low-dose (100 mg) aspirin administration, but not by a higher dose (200–250 mg) given once daily (Dillinger et al., 2012; Pascale et al., 2012). The Aspirin Regimens in Essential Thrombocythemia Study (ARES) is a randomized, parallel-arm, dose-finding study recruiting 300 ET patients to address two main questions (De Stefano et al., 2018). First, whether a bid or tid 100-mg aspirin regimen is more effective than the standard once daily regimen in inhibiting platelet TXA_2_ production, without a major impact on vascular PGI_2_ biosynthesis. Second, whether superior biochemical efficacy of a multiple versus single dosing low-dose aspirin regimen can be safely maintained over long-term follow-up (De Stefano et al., 2018).

Similarly, several independent studies have consistently shown the superior biochemical efficacy of a strategy based on shortening the dosing interval versus a strategy of maintaining or increasing the once daily dose of aspirin in patients with type-2 diabetes mellitus (Spectre et al., 2011; Rocca et al., 2012; Bethel et al., 2016), undergoing CABG (Paikin et al., 2015; Cavalca et al., 2017), or presenting with an acute coronary syndrome (Parker et al., 2019).

**Figure 5:**
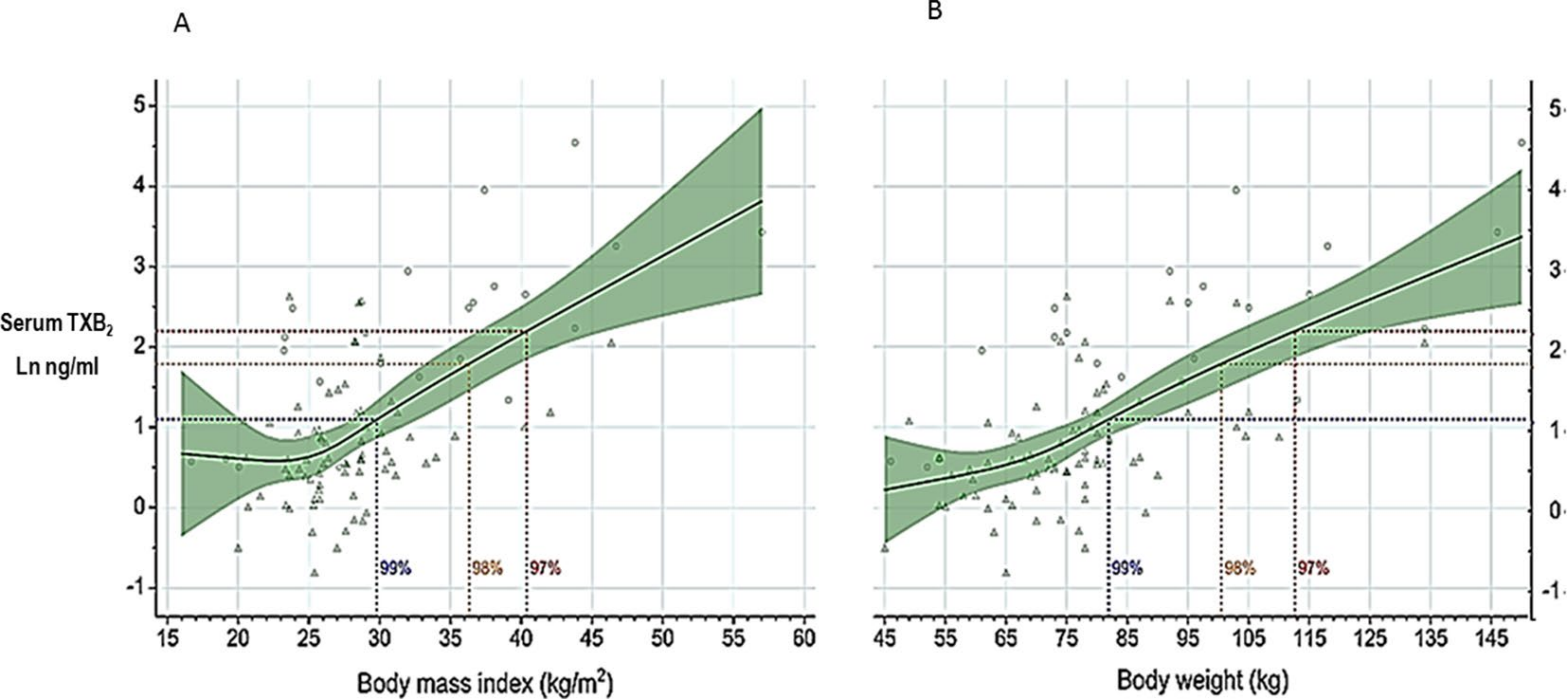
Serum thromboxane (TX)B_2_ levels at 24 h after aspirin intake (100 mg once daily) vs body mass index or body weight. Log-transformed serum TXB_2_ levels measured 24 h after a witnessed aspirin intake are represented in relation to body mass index (panel **A**) and body weight (panel **B**). Triangles represent individual patients receiving aspirin for primary or secondary cardiovascular prevention, n = 71; circles represent individual healthy subjects, n = 25. Solid lines and green areas: predicted serum TXB_2_ levels and 95% confidence intervals, respectively, as a function of increasing body mass index (panel **A**) and body weight (panel **B**) in the 96 study subjects. Horizontal dotted lines were drawn to indicate serum TXB_2_ levels of 3, 6, and 9 ng/mL, corresponding to 99%, 98%, and 97% inhibition of platelet COX-1 activity, respectively, measured in healthy subjects. The vertical dotted lines were drawn to indicate on the abscissa scales the highest calculated values of body mass index (panel **A**) and body weight (panel **B**) compatible with these different levels of platelet COX-1 inhibition. Modified and redrawn from Petrucci et al., 2019, with permission from the publisher.

Obesity is associated with biochemical evidence of persistently enhanced platelet activation (Davì et al., 2002; Petrucci et al., 2019) and high risk of atherothrombotic complications (reviewed by Rocca et al., 2018). Recently, Rothwell et al. (2018) analyzed individual data of 117,279 subjects recruited into 10 primary prevention trials and reported that low doses of aspirin (75–100 mg) were only effective in preventing major vascular events in subjects with body weight lower than 70 kg, and had no benefit in the vast majority of men and nearly 50% of all women weighing 70 kg or more. In contrast, higher doses (≥ 325 mg) of aspirin were only effective in subjects with body weight equal to or higher than 70 kg (Rothwell et al., 2018). Although the finding of effect modification by body weight has not been confirmed by some of the more recent aspirin trials (ASCEND Study Collaborative Group, 2018), these data appear consistent with the suggestion that the antiplatelet effect of aspirin, as reflected by serum TXB_2_ measurement, is influenced by body size (Maree et al., 2005). Increased body size, fat excess, and the associated changes in volume of distribution and liver function may all reduce the bioavailability of a lipophilic drug such as aspirin (Patrono and Rocca, 2017). Petrucci et al. (2019) recently reported measurements of serum TXB_2_ at the end of the 24-h dosing interval in 100 aspirin-treated subjects with a wide range of body mass index (BMI) and body weight values. A statistically significant exponential association was observed between body size, expressed as either BMI or body weight, and residual serum TXB_2_ values (**Figure 5**) (Petrucci et al., 2019). Thus, a standard, once‐daily 100-mg aspirin regimen appears to be inadequate to fully inhibit platelet COX‐1 activity throughout the 24-h dosing interval in moderately to severely obese subjects. However, based on the data depicted in **Figure 5**, one would not expect impaired aspirin pharmacodynamics in subjects with up to a body weight of ∼100 kg or a BMI of 35 kg/m^2^, corresponding to obesity of class 2 or higher (Petrucci et al., 2019). *In silico* modeling of the antiplatelet pharmacodynamics of aspirin in this setting suggested that either doubling the once‐daily dose or administering a lower dose (e.g., 85 mg) twice daily would be associated with adequate suppression of platelet TXA_2_ production(Petrucci et al., 2019). The consensus opinion that “it is reasonable to double the daily dose or shorten the dosing interval (twice-daily) for BMI ≥ 40 kg/m^2^” expressed by the Working Group on Thrombosis of the European Society of Cardiology (Rocca et al., 2018) is consistent with these experimental findings and *in silico* modeling (Petrucci et al., 2019).

## Clinical Implications and Perspective

If we look back at four decades of research on thromboxane biosynthesis and inhibition, the translational aspect of this research stands out in at least three areas. First, measurements of urinary TXM excretion, a non-invasive index of *in vivo* platelet activation, have been instrumental in identifying clinical conditions in which to assess the efficacy and safety of antiplatelet therapy. Notable examples are represented by acute coronary syndromes (Fitzgerald et al., 1986), acute ischemic stroke (Koudstaal et al., 1993), and polycythemia vera (Landolfi et al., 1992). The remarkable cardio-protective effects of low-dose aspirin in these settings (The RISC Group, 1990; Landolfi et al., 2004; Rothwell et al., 2016) are likely to reflect the important pathophysiologic role of transiently or persistently enhanced platelet activation unravelled by TXM measurements. Second, the development of serum TXB_2_ as a mechanism-based biomarker of platelet COX-1 inhibition (Patrono et al., 1980) has played a fundamental role in defining the human pharmacology of aspirin as an antiplatelet agent (Patrono, 1994). The results of a large series of randomized, placebo-controlled clinical trials of aspirin in the prevention of atherothrombosis in high-risk patients have confirmed the saturability of its antithrombotic effect at low doses (Antithrombotic Trialists’ Collaboration, 2002), consistent with saturability of platelet COX-1 inactivation by low-dose aspirin (Patrignani et al., 1982; FitzGerald et al., 1983). Third, a comparison of the extent and duration of serum TXB_2_ reduction in response to different NSAIDs has allowed predicting a similar cardiovascular hazard of tNSAIDs and coxibs (Baigent and Patrono, 2003): “If the vascular consequences of endothelial COX‐2 inhibition are modulated by profound and persistent blockade of platelet COX‐1 activity, as indirectly implied by genetic and pharmacologic manipulations in mice (Cheng et al., 2002), then the cardiovascular effects of most traditional NSAIDs, which only incompletely and transiently inhibit platelet COX‐1, may resemble those of selective COX‐2 inhibitors.” A prediction largely borne out by a tabular data meta-analysis published in 2006 (Kearney et al., 2006) and substantiated by an individual participant data meta-analysis of coxib and tNSAID trials in 2013 (CNT Collaboration et al., 2013).

Finally, as anticipated in the 1982 *Journal of Clinical Investigation* paper, “since the effect of low-dose aspirin is dependent upon platelet turnover as well as aspirin sensitivity of platelet and megakaryocyte cyclooxygenase, the adequacy of this therapeutic regimen might vary in different disease states” (Patrignani et al., 1982). More recent studies based on this methodological approach have largely confirmed this prediction both in specific clinical settings and by *in silico* modelling. Altogether, early and newer evidences have shown the desirability and practicality of adjusting the aspirin dosing interval based on two measurements of serum TXB_2_ at 12 and 24 h after dosing (Pascale et al., 2012; Rocca et al., 2012), a personalized approach that may be required under conditions of reduced systemic bioavailability of aspirin (Petrucci et al., 2019) or enhanced platelet turnover (Patrono et al., 2013).

## Author Contributions

CP has conceived and drafted the work. BR has contributed to the analysis and interpretation of the available literature in the field. CP and BR revised the paper critically for important intellectual content and provided approval for publication of the content.

## Conflict of Interest

CP reports consulting and lecture fees from Acticor Biotech, Amgen, Bayer, GlaxoSmithKline and Zambon, and institutional research grants from Bayer, Cancer Research UK (Catalyst Award—Aspirin for Cancer Prevention Collaboration), European Commission, and Italian Drug Agency (AIFA); he serves as the chairperson of the Scientific Advisory Board of the International Aspirin Foundation. BR reports consulting and lecture fees from Bayer, institutional research grants from Cancer Research UK (Catalyst Award—Aspirin for Cancer Prevention Collaboration), and from the Italian Drug Agency (AIFA).
